# Perceived Effectiveness and Student Satisfaction With Microsoft OneNote for Teaching Respiratory Physiology During the COVID-19 Pandemic: A Cross-Sectional Study Using the Students' Evaluation of Educational Quality Questionnaire

**DOI:** 10.7759/cureus.85006

**Published:** 2025-05-28

**Authors:** Vinu Vij, Jyotsna Gumashta, Smita R Sorte, Gayatri Muthiyan

**Affiliations:** 1 Physiology, All India Institute of Medical Sciences Nagpur, Nagpur, IND; 2 Anatomy, All India Institute of Medical Sciences Nagpur, Nagpur, IND

**Keywords:** medical education, microsoft onenote, online learning, questionnaire, student satisfaction

## Abstract

Introduction: The COVID-19 pandemic necessitated an abrupt transition from in-person to online teaching, presenting significant challenges for medical education. While most educators relied on screen-sharing tools, such as Zoom or Google Meet, to present slide-based content (e.g., PowerPoint), the use of Microsoft OneNote as an interactive instructional interface remains underexplored. This study aimed to assess first-year medical students’ satisfaction and perceived effectiveness of Microsoft OneNote for teaching respiratory physiology.

Methods: A descriptive cross-sectional study was conducted at the All India Institute of Medical Sciences Nagpur (AIIMS Nagpur), Nagpur, India, among first-year Bachelor of Medicine and Bachelor of Surgery (MBBS) students (n = 97) who attended over 90% of synchronous online sessions conducted using Microsoft OneNote alongside video conferencing platforms. A modified version of the validated Students’ Evaluation of Educational Quality (SEEQ) questionnaire was used. Data were analyzed using Statistical Product and Service Solutions (SPSS, version 22.0; IBM SPSS Statistics for Windows, Armonk, NY), applying descriptive statistics and one-way ANOVA.

Results: Students reported high satisfaction in domains such as enthusiasm (mean = 4.61 ± 0.49), individual rapport (mean = 4.46 ± 0.52), and organization (mean = 4.36 ± 0.52). The technology domain scored lower (mean = 3.76 ± 0.47), likely due to connectivity issues. No statistically significant differences in responses were observed across age or gender.

Conclusion: Microsoft OneNote was perceived as an effective and engaging instructional tool for remote physiology teaching. Although the study assessed only perceived effectiveness, the results highlight OneNote’s utility in enhancing instructional clarity, interaction, and content organization. These findings support further exploration of its integration into routine blended medical education settings.

## Introduction

The COVID-19 pandemic disrupted education globally, affecting over 1.6 billion learners across 190 countries [1]. Medical education, being heavily reliant on face-to-face interaction, faced significant challenges during this shift. To address these challenges, faculty members generally adopted web-based video conferencing (WVC) platforms for online instruction. During this transition, the author of this study employed Microsoft OneNote as an instructional interface for teaching respiratory physiology to first-year Bachelor of Medicine and Bachelor of Surgery (MBBS) students.

Microsoft OneNote, part of the Microsoft Office suite, is a digital note-taking application that supports collaborative, real-time interaction. While widely used for personal note management, its educational potential remains underexplored. OneNote offers the ability to write, draw, annotate, and embed multimedia content, simulating traditional blackboard teaching while enabling persistent, revisitable content [2-4].

Used alongside WVC platforms such as Zoom or Teams, OneNote served as the live digital board where lessons were created, annotated, and structured during class. This dual-platform strategy allowed synchronous delivery and interactive engagement while enabling asynchronous review of organized content. Features such as hierarchical folder structure, flexible canvas space, and real-time synchronization across devices offered pedagogical advantages over conventional presentation-based methods [2,3,5].

This study aimed to evaluate student satisfaction and perceived effectiveness of Microsoft OneNote as a digital teaching tool during emergency remote learning, using the validated Students' Evaluation of Educational Quality (SEEQ) questionnaire [6].

## Materials and methods

This descriptive cross-sectional study was conducted among first-year Bachelor of Medicine and Bachelor of Surgery (MBBS) students at All India Institute of Medical Sciences Nagpur (AIIMS), Nagpur, India, who attended synchronous respiratory physiology classes via Microsoft OneNote (Microsoft® Corp., Redmond, WA) from March to May 2021. Ethics approval was obtained from the Institutional Ethics Committee (IEC/Pharmac/2021/272). Written informed consent was obtained.

A modified version of the SEEQ questionnaire was administered. Originally developed by Marsh [6], the SEEQ is a multidimensional student evaluation tool with strong psychometric properties. The original instrument consists of nine dimensions, including learning, enthusiasm, organization, group interaction, individual rapport, breadth, examinations, assignments, and overall rating.

For this study, a version adapted by Arundina et al. [7] for online settings was further modified. Four context-specific questions unrelated to our delivery format were excluded. The final instrument retained 17 items across domains relevant to the online OneNote-based teaching format: learning, enthusiasm, organization, group interaction, individual rapport, technology, and overall satisfaction. Previous studies have reported high internal consistency (Cronbach's alpha = 0.89) for similar adaptations [7].

Data were collected using Google Forms. Responses were scored on a 5-point Likert scale. Data analysis was conducted using Statistical Product and Service Solutions (SPSS, version 22.0; IBM SPSS Statistics for Windows, Armonk, NY). Descriptive statistics (mean, SD, frequencies) and one-way ANOVA were applied to examine associations with demographic variables.

## Results

A total of 97 out of 125 enrolled students completed the questionnaire (response rate: 77.6%). Table 1 summarizes demographic data. Most participants were aged between 18 and 21 years, with nearly equal gender distribution.

**Table 1:** Distribution of the study population according to demographic variables (n = 97)

Independent Variables	Number	Percentage (%)
Age (Years)		
18	12	12.4
19	35	36.1
20	43	44.3
21	7	7.2
Total (Age)	97	100.0
Gender		
Male	47	48.5
Female	50	51.5
Total (Gender)	97	100.0

Table 2 displays response frequencies across SEEQ items. High agreement was noted in enthusiasm (63.9% strongly agreed), individual rapport (59.8%), and learning domains. In contrast, 37.1% of students reported neutral satisfaction in the technology domain, likely due to infrastructural limitations.

**Table 2:** Frequency of responses to SEEQ questionnaire among the study population

Questions	Strongly Disagree n (%)	Disagree n (%)	Neutral n (%)	Agree n (%)	Strongly Agree n (%)
Domain 1: Learning					
1. I have found these Microsoft OneNote sessions intellectually challenging and stimulating.	0	6 (6.2%)	18 (18.6%)	61 (62.9%)	12 (12.4%)
2. I have learned and understood the subject materials of these Microsoft OneNote sessions.	0	0	11 (11.3%)	47 (48.5%)	39 (40.2%)
3. My interest and motivation in learning have increased as a consequence of these Microsoft OneNote sessions.	0	9 (9.3%)	12 (12.4%)	61 (62.9%)	15 (15.5%)
4. The faculty member covered the stated objectives for these Microsoft OneNote sessions.	0	0	7 (7.2%)	51 (52.6%)	39 (40.2%)
Domain 2: Enthusiasm					
5. The faculty member was enthusiastic (excited) about teaching these Microsoft OneNote sessions.	0	0	0	35 (36.1%)	62 (63.9%)
6. The faculty member was dynamic (style of presentation holds your interest) and energetic during these Microsoft OneNote sessions.	0	0	3 (3.1%)	34 (35.1%)	60 (61.9%)
Domain 3: Organization					
7. The faculty member’s explanations were clear.	0	0	5 (5.2%)	40 (41.2%)	52 (53.6%)
8. The Microsoft OneNote sessions material was well prepared and carefully explained.	0	0	5 (5.2%)	40 (41.2%)	52 (53.6%)
9. The faculty member gave a Microsoft OneNote session that facilitated taking notes.	0	4 (4.1%)	10 (10.3%)	54 (55.7%)	29 (29.9%)
Domain 4: Group Interaction					
10. Students were encouraged to participate in these Microsoft OneNote sessions.	0	0	23 (23.7%)	43 (44.3%)	31 (32%)
11. Students were invited to share their ideas and knowledge.	0	3 (3.1%)	18 (18.6%)	47 (48.5%)	29 (29.9%)
12. Students were encouraged to ask questions and were given meaningful answers.	0	0	14 (14.4%)	33 (34%)	50 (51.5%)
Domain 5: Individual Rapport					
13. The faculty member made the students feel welcome in seeking help/advice regarding learning challenges during COVID-19.	0	0	5 (5.2%)	51 (52.6%)	41 (42.3%)
14. The faculty member had a genuine (sincere) interest in students.	0	0	3 (3.1%)	36 (37.1%)	58 (59.8%)
Domain 6: Technology					
15. The faculty member was creative (used whiteboard, chat room, videos, web materials, etc.)	0	0	7 (7.2%)	42 (43.3%)	48 (49.5%)
16. During these Microsoft OneNote sessions, there were technical issues (audio/visual).	5 (5.2%)	19 (19.6%)	36 (37.1%)	35 (36.1%)	2 (2.1%)
Domain 7: Satisfaction					
17. Overall, I was highly satisfied with these Microsoft OneNote sessions.	0	0	8 (8.2%)	52 (53.6%)	37 (38.1%)

Domain-wise mean scores are presented in Table 3. Enthusiasm (4.61 ± 0.49), individual rapport (4.46 ± 0.52), and organization (4.36 ± 0.52) received the highest ratings. One-way ANOVA revealed no statistically significant differences in scores by age or gender (p > 0.05).

**Table 3:** Comparative assessment of domains of the SEEQ questionnaire according to age and gender SEEQ: Students' Evaluation of Educational Quality

Demographic Variables	Learning (Mean ± SD)	Enthusiasm (Mean ± SD)	Organization (Mean ± SD)	Group Interaction (Mean ± SD)	Individual Rapport (Mean ± SD)	Technology (Mean ± SD)	Satisfaction (Mean ± SD)
Age (years)							
18	4.16 ± 0.55	4.62 ± 0.48	4.41 ± 0.42	4.47 ± 0.55	4.58 ± 0.46	3.75 ± 0.39	4.50 ± 0.52
19	3.97 ± 0.53	4.50 ± 0.54	4.21 ± 0.52	4.03 ± 0.60	4.31 ± 0.55	3.72 ± 0.37	4.11 ± 0.58
20	4.11 ± 0.49	4.70 ± 0.43	4.46 ± 0.50	4.24 ± 0.67	4.58 ± 0.44	3.80 ± 0.56	4.40 ± 0.62
21	4.11 ± 0.69	4.57 ± 0.61	4.33 ± 0.72	3.85 ± 0.89	4.35 ± 0.74	3.71 ± 0.48	4.29 ± 0.75
p-value	0.587	0.328	0.221	0.114	0.115	0.908	0.138
Gender							
Male	4.12 ± 0.48	4.59 ± 0.47	4.35 ± 0.45	4.18 ± 0.65	4.48 ± 0.46	3.84 ± 0.39	4.32 ± 0.55
Female	4.02 ± 0.57	4.63 ± 0.52	4.36 ± 0.58	4.15 ± 0.67	4.45 ± 0.58	3.69 ± 0.53	4.28 ± 0.67
p-value	0.346	0.737	0.911	0.819	0.714	0.119	0.756
Total	4.06 ± 0.53	4.61 ± 0.49	4.36 ± 0.52	4.16 ± 0.66	4.46 ± 0.52	3.76 ± 0.47	4.30 ± 0.61

**Figure 1:**
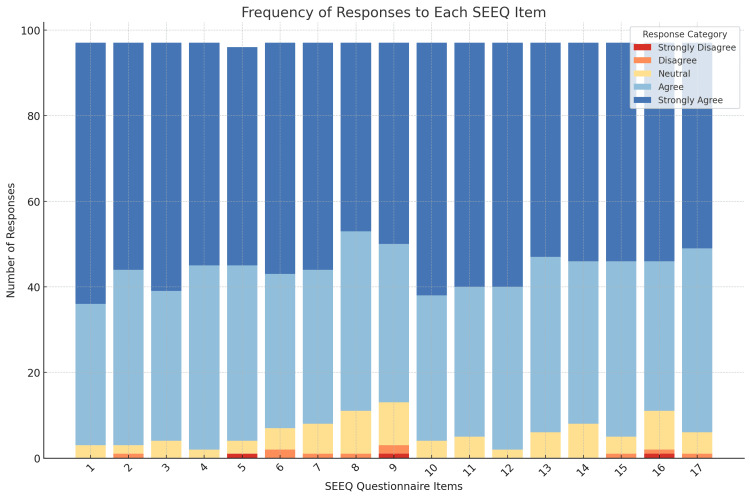
Frequency of responses across all 17 SEEQ items SEEQ: Students' Evaluation of Educational Quality

Figure 1 depicts the frequency of responses across all 17 SEEQ items. It visually reinforces the positive response trends, especially in teaching-related domains.

## Discussion

The findings of this study indicate that Microsoft OneNote, when used in conjunction with a WVC platform, was perceived as a satisfactory and engaging tool for delivering online instruction during the pandemic-imposed shift to remote teaching. The use of OneNote offered an enriched visual and interactive environment that facilitated the delivery of complex physiological concepts. High levels of satisfaction reported by students, particularly in domains such as instructor enthusiasm, individual rapport, and organization, align with the indicators of teaching presence as outlined in the Community of Inquiry (CoI) framework [4].

The CoI framework posits that successful educational experiences in online learning environments depend on the interplay of three core elements: teaching presence, cognitive presence, and social presence. OneNote’s persistent and organized digital canvas supported structured content delivery and annotation-based teaching, enhancing teaching presence. Furthermore, features such as collaborative editing, embedded media, and real-time annotations encouraged student participation, thereby promoting cognitive and social presence.

Interestingly, the technology domain received relatively lower satisfaction scores compared to others. Approximately 36.1% of students agreed that technical problems occurred during the sessions, and 37.1% expressed neutrality. These findings reflect infrastructural limitations such as internet instability and device incompatibility, which are often cited as barriers in low-resource settings [7,8]. Nevertheless, OneNote’s features, such as auto-save, cross-device syncing, and asynchronous access, helped mitigate some of these limitations by allowing students to revisit lecture content at their convenience.

Compared to conventional presentation tools, such as PowerPoint, OneNote enabled spontaneous content generation and live annotation, closely mimicking the dynamics of a blackboard lecture. This real-time flexibility is particularly useful in subjects such as physiology that require diagrammatic explanations, sequential illustrations, and iterative clarifications. Furthermore, unlike platforms limited to video streaming, OneNote offers a persistent, editable content structure that remains accessible even after the session ends. This contributed to high ratings in domains such as organization and learning.

In addition to CoI, the Substitution Augmentation Modification Redefinition (SAMR) model offers a relevant pedagogical lens for evaluating OneNote's integration into online teaching [6]. OneNote appears to move beyond mere substitution of traditional tools by enabling augmentation (multimedia content), modification (interactive note-sharing), and potentially redefinition (student-created content spaces). This transition from enhancement to transformation aligns with constructivist teaching approaches and supports deeper learning.

Moreover, previous studies emphasize that, while perceived satisfaction does not equate to measurable learning outcomes, it correlates with enhanced motivation, engagement, and long-term retention [5]. The SEEQ instrument used in this study, with a validated structure and high internal consistency, offered a comprehensive evaluation of perceived effectiveness across multiple pedagogical domains [2,3]. The authors’ justified modification of the SEEQ - excluding context-specific and open-ended items - preserved psychometric robustness while enhancing contextual relevance.

Our findings resonate with prior literature that demonstrates the effectiveness of intentional digital tool integration in maintaining instructional quality during crisis scenarios [8,9]. Although this study focused on respiratory physiology, the pedagogical benefits observed with OneNote suggest its potential applicability across other subjects in medical education. Its utility lies not merely in content delivery but in shaping the digital learning environment to support interactivity, clarity, and accessibility.

However, this study is not without limitations. The absence of a control group or objective performance measures limits conclusions regarding actual learning gains. Additionally, while OneNote’s functionality was leveraged effectively by the author, user experience may vary depending on the instructor’s familiarity with the platform. Future studies could explore longitudinal learning outcomes, compare OneNote with other platforms, or incorporate qualitative data to enrich understanding of student perspectives.

Overall, Microsoft OneNote emerges as a promising adjunct in remote and blended learning contexts, particularly for subjects that benefit from visual explanation, sequential logic, and synchronous engagement. Its integration into pedagogical practice - when guided by established educational frameworks - can support dynamic, learner-centered instruction even in challenging teaching environments.

## Conclusions

Microsoft OneNote, when used in tandem with web conferencing platforms, emerged as a robust and highly satisfactory teaching tool for delivering respiratory physiology content in an online format. The positive student perceptions, particularly in domains such as instructor enthusiasm, individual rapport, and instructional organization, underscore the platform’s ability to enhance teaching presence - a critical component in the CoI framework. These findings suggest that OneNote not only supports effective content delivery but also fosters real-time interactivity, structured visualization, and persistent access to materials, all of which contribute to deeper learner engagement.

While this study focused on respiratory physiology, the pedagogical strengths of OneNote are not discipline-specific. Its adaptability, multimodal content integration, and collaborative features make it a compelling adjunct for a wide range of subjects in medical education. The high satisfaction scores reflect its perceived value during emergency remote teaching, and they warrant further exploration of its role in structured blended learning models. Future research should focus on comparative analyses, integration with learning management systems (LMS), and evaluation of long-term academic outcomes to substantiate its utility beyond perception-based metrics. In summary, OneNote represents a promising digital platform that bridges traditional and modern pedagogy. Its integration into regular curricula can enrich instructional delivery, promote learner autonomy, and enhance the overall educational experience in both crisis-driven and conventional academic settings.
